# Dentinogenesis imperfecta in Osteogenesis imperfecta type XI in South Africa: a genotype–phenotype correlation

**DOI:** 10.1038/s41405-019-0014-z

**Published:** 2019-04-11

**Authors:** M. Chetty, T. Roberts, S. Shaik, P. Beighton

**Affiliations:** 1 Faculty of Dentistry, University of the Western Cape, Bellville, Cape Town, South Africa; 2 University of the Western Cape/University of Cape Town Collaborative Dental Genetics Clinic, Red Cross Children’s Hospital, Cape Town, South Africa; 3 Faculty of Health Sciences, Division of Human Genetics, University of Cape Town, Rondebosch, South Africa

## Abstract

**Background:**

The maxillofacial and dental manifestations of Osteogenesis imperfecta (OI) have significant implications in terms of management. Although the occurrence of abnormal dentine in some forms of OI is well documented, there is scant information on the association of abnormal dentine in the Black African persons with phenotypic OI III and genotypic OI XI in South Africa.

**Methods:**

This was a cross-sectional analytic study. A series of 64 Black South African individuals with a confirmed phenotypic diagnosis of OI III, ages ranging from 3 months to 29 years, were assessed clinically, radiographically, and at a molecular level.

**Results:**

A total number of 64 saliva samples were analyzed and 3 DNA variations were identified in exon 5 of the *FKBP10* gene. The homozygous mutation, c.[831dupC]; [831dupC], was identified in 23 affected persons who had no clinically obvious features of DI in their primary and secondary teeth. Radiologically, mild features of DI were evident in 10 persons in whom radiographic images were obtained and were given a Clinical–radiological score of 2. A compound heterozygous mutation, c. [831delC]; [831dupC], was identified in three siblings. An intraoral examination of these affected persons revealed no clinically apparent features of DI in their primary and secondary teeth. Due to the lack of radiological facilities, the presence or absence of DI could not be confirmed or negated. A second compound heterozygous mutation, c.[831dupC]; [1400-4C>G], was identified in a female of 29 years belonging to the Xhosa linguistic group. Her teeth appeared clinically normal but it was not possible to obtain radiographs. In 37 affected individuals, no disease-causing mutations were identified.

**Conclusion:**

Black African individuals in SA with the homozygous mutation in the *FKBP10* gene have clinically unaffected teeth yet exhibited radiographic features of DI to varying degrees. This characterization is suggestive of a relationship between the genetic abnormality and the clinical manifestations of DI. The authors suggest that this diagnosis must include teeth that are clinically and/or radiologically aberrant, and should not exclude the presence of other, milder, dentinal aberrations associated with OI. There was no correlation between severity of OI and DI in this cohort of individuals.

## Introduction

Osteogenesis imperfecta (OI) is a heterogeneous group of disorders in which skeletal fragility and frequent fractures are the major features. The classification and nosology of OI has been through a number of revisions reflecting the discovery of new subtypes, initially as clinical entities and subsequently defined by their molecular aetiology. These molecular findings have resulted in an expanded classification of OI from Types I – XV, but, it has been difficult to maintain correlations between the Sillence types and their molecular basis. This situation prompted the International Nomenclature Committee for Constitutional Disorders of the Skeleton in 2015^1^ to propose a return to a clinical classification of five subtypes, as proposed by Van Dijk and Sillence (2014)^2^ and illustrated in Table 1.

**Table 1:** Current OI nomenclature and associated modes of inheritance^2^

Name of syndrome	Inheritance mode	Equivalent numerical types
Classic non-deforming OI with blue sclerae (OI type 1)	AD	I
Peri-natally lethal OI (OI type 2)	AD, AR	II
Progressively deforming OI (OI type 3)	AD, AR	III
Common variable OI with normal sclerae (OI type 4)	AD, AR, XL	IV
OI with calcification in interosseous membranes (OI type 5)	AD	V

The nosological relationship between OI III and OI XI has potential for confusion. In essence, OI III was defined at a clinical and radiological level by Sillence et al.^3,4^ Further subdivision of the established forms of OI followed the elucidation of the biochemical defect in collagen in a small number of affected persons. Thereafter, some persons with the OI III phenotype were found to have a mutation in the *FKBP10* gene. These individuals were classified as having OI XI, which is defined as an autosomal recessive form of OI caused by a homozygous mutation in the *FKBP10* gene in chromosome 17q21.^5^

In South Africa and Zimbabwe, a severe form of OI III has been found to be fairly common in the indigenous Black African population.^6^ This condition is characterized by numerous fractures, gross deformity of tubular bones, spinal malalignment, and marked impairment of growth. Multiple fractures may be present at birth, but the specific radiological appearances in the newborn have not yet been documented. Physical handicap in severe and affected children often become wheelchair bound. Death before adulthood is frequent. Affected individuals have been recognized in the Sotho, Pedi, Swazi, Zulu, and Tswana linguistic groups among others and an overall ratio of OI I to OI III as 1–6 was estimated in this population group.^7^ It was suggested that the reason for this high prevalence is that the unaffected heterozygote may have a biological advantage in the African environment and that the mutation for OI III in Africa occurred more than 2000 years ago in West or Central Africa prior to migration to present day Southern Africa.^7^

Although worldwide, autosomal recessive OI is rare, the frequency of a form of OI III is relatively high in the indigenous Black African population of South Africa.^6–8^ When molecular investigatios have been undertaken, mutations in the *FKBP10* gene that encodes the collagen chaperone-like protein FKBP65 were identified.^9^ *FKBP10* is one of the newer members of an expanding list of AR OI genes and was first documented in OI III by Alanay et al.^10^

The group of patients described in the current study have an AR type of phenotypic OI III^6–12^ and the *FKBP10* genotype which thereby modified the diagnosis to that of OI XI.

The occurrence of abnormal dentine in some forms of OI is well documented, however, there is scant information on the association of phenotypic OI III and genotypic OI type XI with abnormal dentine in South Africa(SA) in the Black African population. The aim of this study was to document the status of DI in persons with the OI XI genotype in South Africans.

## Methods

This was a cross-sectional analytic study. The dental findings which form the subject of this article were derived from a series of 64 Black South African individuals with a confirmed phenotypic diagnosis of OI III, ages ranging from 3 months to 29 years, who were assessed clinically, radiographically, and at a molecular level. This investigation was a component of a PhD investigation by the researcher (MC) at the University of Cape Town.

This study had a predominant clinical component in which dental and craniofacial abnormalities in the affected persons were documented. Although radiographic resources were limited, 15 CBCT images, 20 panorex, and 20 cephalometric radiographs were obtained. All radiographic findings were confirmed by two consultant radiologists.

Biological material from these affected persons was analyzed for a mutation in exon 5 of the *FKBP10* gene which has the cytogenetic location 17q21.2. The Oragene saliva collection kits were used and DNA extracted from epithelial cells was examined.

In order to consolidate the clinical and radiological information, a clinical–radiological score (CRS), developed by Scandinavian authors, was employed.^13^

### Clinical–Radiological Score (CRS)

(1) No clinical or radiographic signs of DI

(2) Only subtle clinical and/or radiological signs of DI

(3) Either obvious clinical or radiological signs of DI

(4) Clear clinical and radiographic signs of DI

In the Scandinavian study (2003), 52 persons with OI were examined and exfoliated or teeth extracted for orthodontic purposes were obtained from all individuals and analyzed histologically for signs of dysplastic dentin. Teeth from 20 unaffected control individuals were also examined. There was a statistical difference in the lower dysplastic dentin score in healthy controls individuals and those with OI and no apparent DI. The higher dysplastic dentin score correlated with a higher CRS.^13^

Following the perusal of the literature^14–17^ the authors propose that a spectrum of changes, which includes taurodontism, can be identified depending on the severity of the condition. The following list has been formulated which highlights the radiological features of dysplastic dentin:

- Crown dysmorphology (bulbous crowns to mild occlusal abnormalities)
- An accentuated constriction at the cemento-enamel junction
- Variable obliteration of the pulp chambers (narrowed roots to abnormally large root canals)
- Taurodontism
- Periapical radiolucency with or without pulp exposure

All investigations were undertaken in complete accordance with the Declaration of Helsinki, the Hippocratic Oath, and the Singapore Statement on Research Integrity. Formal ethical approval (HREC reference number: 203/2013) was obtained from the University of Cape Town’s ethics committee. Informed consent was obtained for all relevant investigations and publication.

## Results

Representative photographic and radiographic images of individuals in each molecular category are presented in order to highlight the findings in terms of DI and attempt to elicit a possible genotype–phenotype correlation. In the images, affected individuals are represented by alphabetical–numerical designations pertaining to the investigation center and the chronological order in which they were assessed.

A total number of 64 saliva samples were analyzed and 3 DNA variations were identified.

**Fig. 1:**
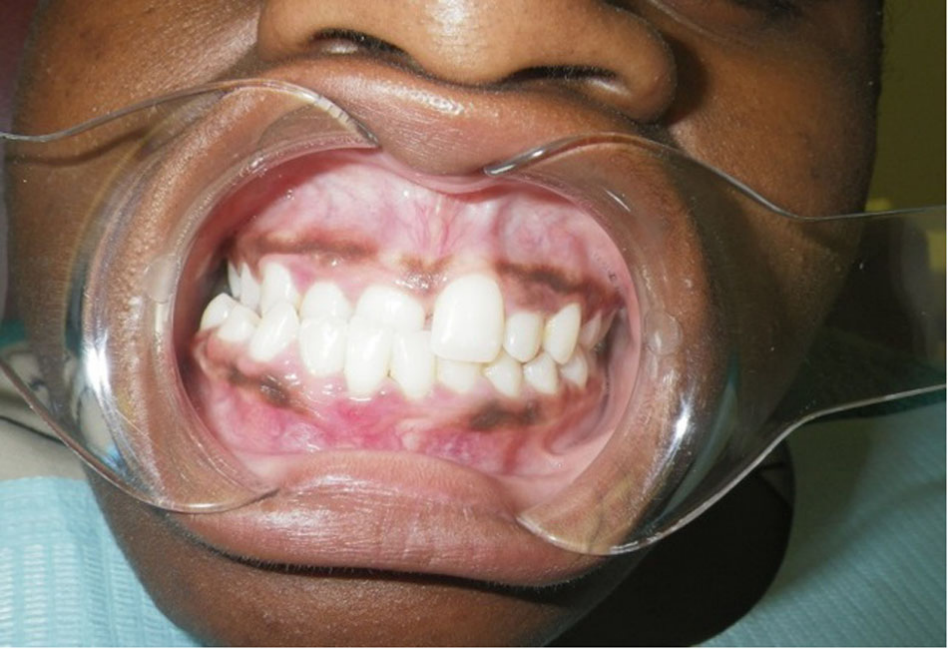
An intraoral picture of CPT 1 shows apparently normal teeth. She gave no history of discolored primary teeth

1. The homozygous mutation was identified in 23 affected persons. This homozygous mutation, c.[831dupC]; [831dupC], a frameshift DNA variant which is predicted to alter the protein sequence by substituting a Glycine residue with an Arginine at position 278 of the 65 kDa FK506 Binding protein 10. The introduction of a premature termination codon results in the loss of 211 amino acid residues.
  No clinically obvious features of DI were evident (Fig. 1) in the primary and secondary teeth of all the 23 individuals. Radiologically, mild features of DI were evident in 10 persons in whom radiographic images were obtained (Fig. 2). These *FKBP10* (HOM) individuals displayed clinically normal teeth but after radiographic examination were given a CRS of 2.

**Fig. 2:**
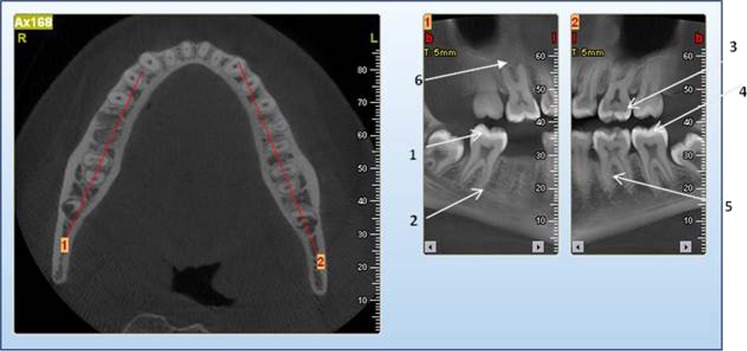
Cropped CBCT image of Individual CPT 1 with features of mild DI which include hypotaurodontism (1), thin short roots of molars (2), cervical constriction of crowns of molars (3), abnormal occlusal anatomy (4), and focal areas of narrowed and occluded root canals (5). Periapical radiolucencies involving teeth 17 and 27 (6)
2. A compound heterozygous mutation, c. [831delC]; [831dupC] was identified in three siblings. This is a frameshift DNA variant in exon 5 of *FKBP10* which is predicted to alter the protein sequence by substituting a Glycine residue with an Alanine at position 278 of the 65 kDa FK506 Binding protein 10. A premature termination codon results in the loss of 286 amino acid residues.
  An intraoral examination of these affected persons revealed no clinically apparent features of DI in their primary and secondary teeth. Due to the lack of radiological facilities, the presence or absence of DI could not be confirmed or negated (Figs. 3 and 4).

**Fig. 3:**
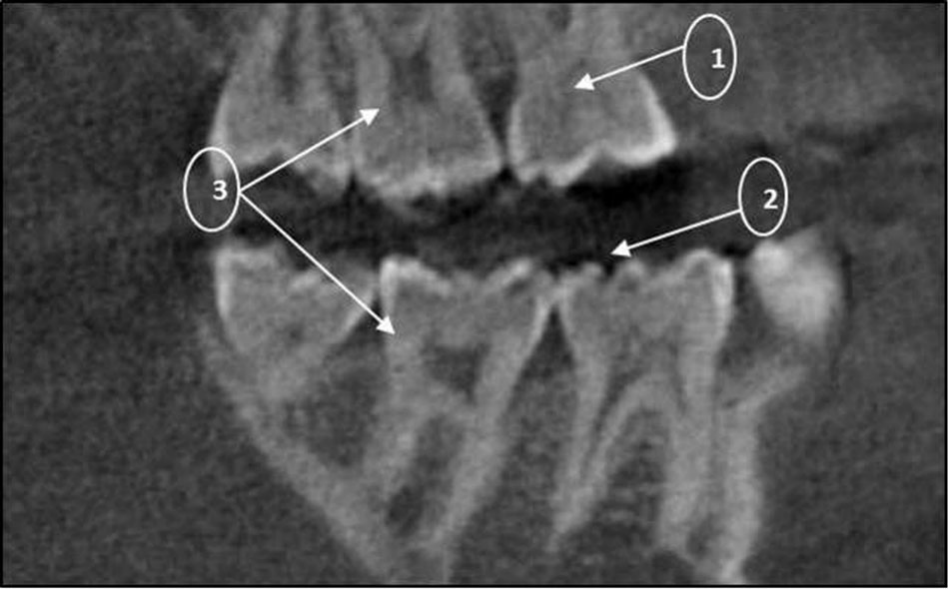
Cropped CBCT image of DBN 4 showing intrapulpal calcifications (1) as well as an unusual occlusal anatomy (2). Features of hypotaurodontism are evident in the 17 and 27 (3)

**Fig. 4:**
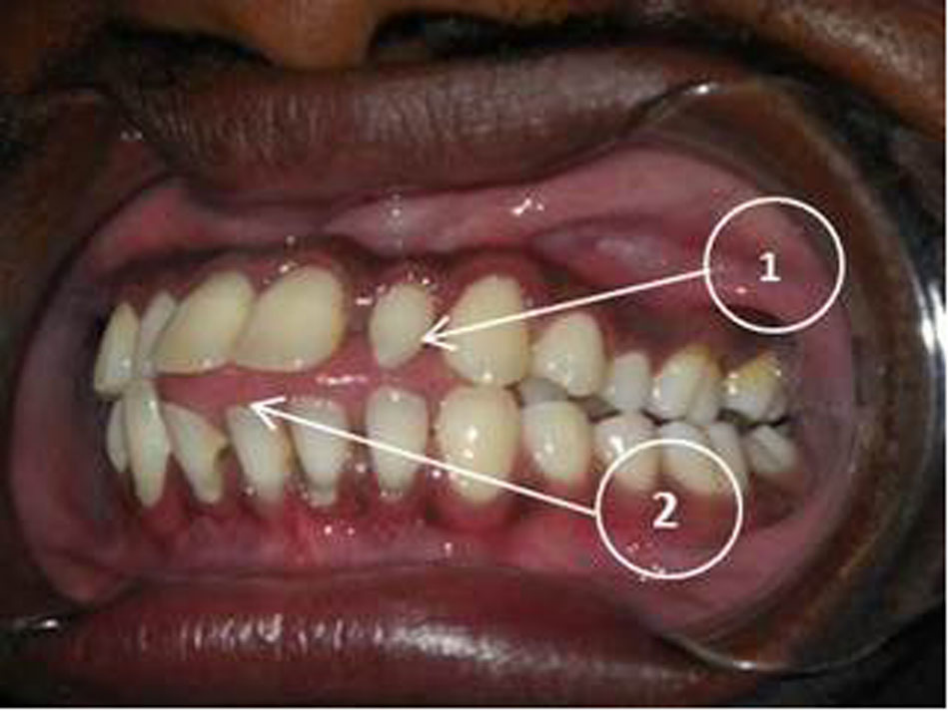
An intraoral picture (QQ2) shows no abnormal discolouration of his teeth. A peg shaped lateral incisor (1) and an anterior open bite (2) are evident
  Both the of the above frameshift variations result in the loss of two peptidylprolyl isomerase domains (PPIase 3 and PPIase 4) and both EF-hand (EF-hand 1 and EF-hand 2) domains which are essential for localization to the endoplasmic reticulum.
3. A second compound heterozygous mutation, c.[831dupC]; [1400-4C>G] was identified in a female of 29 years (Fig. 5) belonging to the Xhosa linguistic group. The designation ‘c.1400-4c>g’, means that a single nucleotide has been substituted 4 bases from the intron–exon junction. This mutation does not change the protein coding sequence directly, it alters the splicing of the exon that it precedes.

**Fig. 5:**
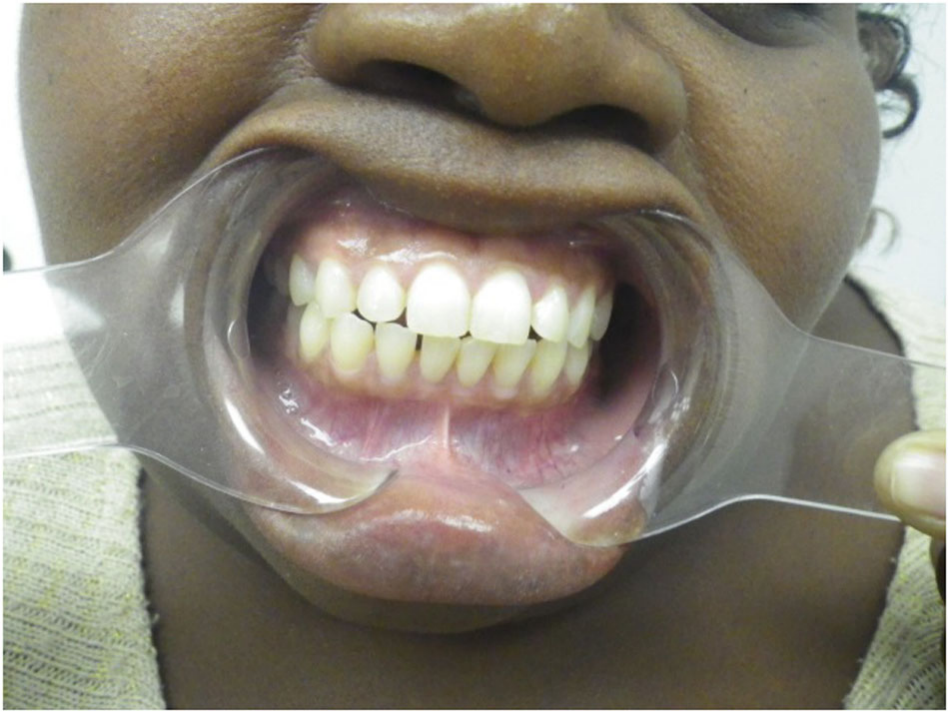
Her (CPT7) anterior teeth are mildly proclined
  Radiographs proved impossible to obtain due to her physical deformity. Her maximum oral opening was 30 mm and bitewing radiographs also proved challenging to obtain. Her teeth appeared clinically normal (Fig. 5).
4. The 37 affected individuals in whom no disease-causing mutations have been identified in exon 5, will be utilized for ongoing mutation screening of the remaining coding regions of *FKBP10* in an attempt to account for all the genetic determinants in this patient cohort. In order to maintain the focus and clarity of this report, no further reference is made to this group.

## Discussion

As phenotypic OI III is genetically heterogeneous, the results of the molecular investigation enabled possible genotype–phenotype correlations at the dental level in the context of this project.

The *FKBP10* gene is located on chromosome 17 from base pair 41,812,261 to base pair 41,823,216.

This gene encodes the FK506-binding protein 65 (FKBP65) found in the endoplasmic reticulum. FKBP65 is a chaperone protein which is important for the processing of collagen and elastin, which are components of the extracellular matrix (ECM). In the ECM, FKBP65 binds to the collagen molecule through a hydroxylation reaction that modifies a particular region of the molecule and enables the correct folding. It also promotes thermal stability of the collagen triple helix, and cross-link formation between collagen molecules to form fibrils.^10^ These modifications to the collagen molecule need to take place in an orderly and timely sequence and chaperone proteins such as FKBP65 regulate this process.^18^ In this context, FKBP65 encoded by *FKBP10* acts as a molecular chaperone for type I procollagen.^2^

The homozygous mutation, c.[831dupC]; [(831dupC)], which was predominant in this project has been reported 19 times on the Leiden Open Variation Database. However, to the best of the authors knowledge, only nine individuals with this homozygous mutation have been documented in published reports.^12,19–21^ The phenotypic features of these nine persons ranged from progressively deforming OI to Bruck Syndrome (BS). Bruck syndrome is an autosomal recessive disorder characterized by the combination of OI III and pterygium formation across large joints with resultant contractures and marked reduction in mobility.^22^ The condition is genetically heterogeneous with BS type 1 caused by mutations in *FKBP10* and BS type 2, which is rare, caused by a mutation in *PLOD2*. These reports were orientated to the discussion of BS type 1 and 8 of these affected persons had contractures. The teeth were described as normal in each of the nine affected persons. This observation concurs with the findings in this South African study where the 23 individuals with the *FKBP10* HOM c.[831dupC]; [831dupC] mutation showed no clinical features of DI in their primary and secondary dentition, but radiographically displayed features of mild DI.

Individuals with the compound heterozygous mutation in the *FKBP10* gene also had teeth which appeared clinically unaffected but with radiographic evidence of DI. These observations are suggestive of a genotypic–phenotypic relationship in terms of the manifestation of DI.

Chaperone proteins such as FKBP65 regulate the complex modifications of the collagen molecule specifically, correct folding, thermal stability of the triple helix, and cross-link formation between collagen molecules.^18^ Therefore, the abnormality in the dentine is due to the irregularity in the collagen matrix. Several studies indicate that DI at clinical, radiological, and histological levels is more frequent in clinical OI III and OI IV.^3,23,24^ Conversely, the clinical dental findings in the South African(SA) cohort of 23 individuals showed phenotypically normal teeth but had radiological features of abnormal dentine. This is most likely the explanation for previous reports of ‘normal teeth’ documented in the SA persons with phenotypic OI III.^6^ Several studies have shown that teeth with no apparent clinical DI in association with OI, do have radiological, histological, and electron microscopic abnormalities of dentine.^13,25,26^ On the basis of these findings, it is reasonable to propose that discolouration of teeth should not be the minimum criterion for the diagnosis of DI.^27^

A Scandinavian study (2003) of 52 persons with OI were examined and exfoliated or teeth extracted for orthodontic purposes were obtained from all individuals and analysed histologically for signs of dysplastic dentin. Teeth from 20 unaffected control individuals were also examined. In this investigation, there was a statistical difference in the lower dysplastic dentin score in healthy controls individuals and those with OI and no apparent DI. The higher dysplastic dentin score correlated with a higher CRS.^13^

In the present SA survey, it was apparent that the ‘normal’ teeth in affected individuals do have a degree of dysplastic dentin. The findings of this project in terms of DI are consistent with the observations of the above-mentioned Scandinavian study and these authors further concluded that the degree of dysplastic dentin correlated with severity of the disorder and indicated that the degree of dentin dysplasia highest in their cohort of OI III affected persons.^13^ Conversely, the findings of this SA project do not show a correlation between the severity of the condition and the degree of dentin dysplasia. The subtle CRS changes evident in the dentin of persons with the *FKBP10* (HOM) positive mutations are most probably an expression of genetic and epigenetic factors which are associated with OI XI in South Africa.

Another study^2^ demonstrated that *FKBP10* mutations not only cause Osteogenesis imperfecta type III but can also result in a severe type of isolated Osteogenesis imperfecta type IV with prenatal onset. These authors also add DI to the spectrum of clinical symptoms associated with *FKBP10* mutations, which is contrary to the findings in the South African group of patients.

OI is a genetically heterogeneous disorder and it is the consequence of mutations in the determinant gene. The abnormality is expressed in the resultant collagen molecules and the ensuing phenotypic features are a reflection of this intragenic heterogeneity.

## Conclusion

Black African individuals in SA with the homozygous and compound heterozygous mutations in the *FKBP10* gene have clinically unaffected teeth yet exhibited radiographic features of DI to varying degrees. This characterization is suggestive of a relationship between the underlying genetic abnormality and the clinical manifestation of DI. The authors suggest that this diagnosis of DI must include teeth that are clinically and/or radiologically aberrant, and should not exclude the presence of other, milder, and dentinal aberrations associated with OI. There was no correlation between severity of OI and DI in this cohort of individuals.
